# Psychiatric disorders and military convalescence: Prevalence, duration of inactivity and operational impact

**DOI:** 10.1192/j.eurpsy.2025.1834

**Published:** 2025-08-26

**Authors:** N. Khaterchi, S. Kamoun, H. Ziedi, A. Amri

**Affiliations:** 1 Occupational Medicine, Military Occupational health center, tunis, Tunisia

## Abstract

**Introduction:**

Military personnel are exposed to intense stress and potentially traumatic environments, making them particularly vulnerable to psychiatric disorders. These pathologies can lead to prolonged periods of convalescence, affecting not only the health of individuals, but also the operational readiness of the armed forces.

**Objectives:**

To identify the most common psychiatric causes of convalescence in military personnel and assess their impact on the operational readiness of the armed forces.

**Methods:**

This was a retrospective study of a descriptive nature, from January 1, 2023 to August 30, 2024, which focused on military personnel who presented a request for convalescence to the occupational pathology consultation at the Military Center for Occupational Medicine and Professional Safety in Tunis.

**Results:**

During the study period, 275 patients were included in the sample, with a mean age of 40.83 ± 9.93 years and a sex ratio of 2.77. Their average length of service was 15.62 ± 11.87 years. Depression was the most frequent psychiatric pathology, accounting for 70.9% of convalescence causes, followed by anxiety disorder at 8.7%. The most affected category was non-commissioned officers, accounting for 54.7% of cases. The ranks most affected were warrant officer in 12% of cases, chief sergeant in 11.6% of cases and head warrant officer in 10.9% of cases. Military healthcare and administration were the most represented specialties, in 17.5% and 14.2% of cases respectively. The most common workstations were nurses (15.6% of cases), administrative officers (12.4%) and infantrymen (6.2%). The average length of convalescence was 74.78 ± 88.77 days. Convalescence lasting more than 180 days accounted for 10.3% of cases.

**Conclusions:**

Our study shows that psychiatric pathologies have a significant impact on operational readiness. The often prolonged periods of convalescence highlight the importance of implementing effective prevention and management strategies to mitigate the impact of psychiatric disorders within the armed forces.

**Disclosure of Interest:**

None Declared

